# Research on Mental Stress Recognition of Depressive Disorders in Patients With Androgenic Alopecia Based on Machine Learning and Fuzzy K-Means Clustering

**DOI:** 10.3389/fgene.2021.751791

**Published:** 2021-11-12

**Authors:** Yulong Li, Baojin Wu, Xiujun Li, Qin Zhou, Xin Yang, Yufei Li

**Affiliations:** ^1^ Department of Military Medical Psychology, Air Force Medical University, Xi’an, China; ^2^ Department of Plastic Surgery, Huashan Hospital, Fudan University, Shanghai, China; ^3^ College of Education, Shanghai Normal University, Shanghai, China; ^4^ Department of Plastic Surgery, Shanghai East Hospital, School of Medicine, Tongji University, Shanghai, China; ^5^ Medical Cosmetic Center, Department of Dermatology, Tongji Hospital, School of Medicine, Tongji University, Shanghai, China

**Keywords:** meta heuristic algorithm, androgenetic alopecia, depressive disorder, psychological intervention, machine learning and fuzzy K-means clustering

## Abstract

Under the new trend of industry 4.0 software-defined network, the value of meta heuristic algorithm was explored in the recognition of depression in patients with androgenic alopecia (AGA), and there was an analysis on the effect of comprehensive psychological interventions in the rehabilitation of AGA patients. Based on the meta heuristic algorithm, the Filter and Wrapper algorithms were combined in this study to form a new feature selection algorithm FAW-FS. Then, the classification accuracy of FAW-FS and the ability to identify depression disorders were verified under different open data sets. 54 patients with AGA who went to the Medical Cosmetic Center of Tongji Hospital were selected as the research objects and rolled into a control group (routine psychological intervention) and an intervention group (routine + comprehensive psychological interventions) according to different psychological intervention methods, with 27 cases in each group. The differences of the self-rating anxiety scale (SAS), self-rating depression scale (SDS), Hamilton depression scale (HAMD), and physical, psychological, social, and substance function scores before and after intervention were compared between the two groups of AGA patients, and the depression efficacy and compliance of the two groups were analyzed after intervention. The results showed that the classification accuracy of FAW-FS algorithm was the highest in logistic regression (LR), decision tree (DT), K-nearest neighbor (KNN) algorithm, support vector machine (SVM) algorithm, and random forest (RF) algorithm, which was 80.87, 79.24, 80.42, 83.07, and 81.45%, respectively. The LR algorithm had the highest feature selection accuracy of 82.94%, and the classification accuracy of depression disorder in RF algorithm was up to 73.01%. Besides, the SDS, SAS, and HAMD scores of the intervention group were lower sharply than the scores of the control group (*p* < 0.05). The physical function, psychological function, social function, and substance function scores of the intervention group were higher markedly than those of the control group (*p* < 0.05). In addition, the proportions of cured, markedly effective, total effective, full compliance, and total compliance patients in the intervention group increased obviously in contrast to the proportions of the control group (*p* < 0.05). Therefore, it indicated that the FAW-FS algorithm established in this study had significant advantages in the recognition of depression in AGA patients, and comprehensive psychological intervention had a positive effect in the rehabilitation of depression in AGA patients.

## Introduction

Androgenetic alopecia (AGA) is a kind of hair loss skin disease which is characterized by non-scarring and progressive hair follicle miniaturization. It is a common clinical skin disease, mainly characterized by shortening of hair follicle growth period, terminal hair follicle miniaturization, and progressive thinning of hair. The incidence of AGA was approximately 50% in white males over 40 years old and 32.2% in white females over 20 years old (Lolli et al., 2017). Psychosocial factors may aggravate or recur the condition of patients, and patients with hair loss are more likely to suffer from various physical and mental disorders such as anxiety and depression than normal people (Starace et al., 2020). Studies have shown that negative emotions such as anxiety and depression lead to a decline in the ability to deal with challenges and solve problems in patients with hair loss, which seriously affects their quality of life (Tanaka et al., 2018). A large number of investigation reports and meta-analysis prompts to inquire about the cognitive evaluation, emotional expression, and response of different patients to their diseases. Moreover, they have received the necessary psychological interventions, so as to establish scientific disease cognition and psychological behavioral responses. Its own adjustment is employed to promote patient adaptability and disease outcome, which is more significant than treating the disease itself (Rajabi et al., 2018; Völker et al., 2020).

Big data based on Industry 4.0 has the characteristics of large capacity, low signal-to-noise ratio, multiple types, high latitude, and fast access speed. Therefore, there are obvious differences in the methods of identification, analysis, and mining for industrial big data (Lake, 2019). Electroencephalogram (EEG) plays an important role in the diagnosis and recognition of depression. Deep learning can learn useful EEG signals automatically from the original data, to perform pattern recognition process, especially suitable for brain electrical signal recognition task. Many researchers will combine the deep learning algorithm with EEG, to operate EEG with feature extraction, selection, and classification, which can provide an auxiliary tool for the clinical diagnosis of depression (Craik et al., 2019). What’s more, meta heuristic algorithm is a combination of random algorithm and local search algorithm, which is featured with self-organization, self-adaptation, and self-learning. It has been extensively applied in image recognition and classification (Munoz et al., 2018), and it has been also adopted in the recognition of depression (Phadikar et al., 2021). Eilbeigi et al. (2018) , (Eilbeigi and Setarehdan, 2018) used meta-heuristic algorithm to classify EEG data of patients with depression, with the highest accuracy of 78.24%. However, most of the current deep learning methods for depression recognition are to manually extract multiple features and simply combine the extracted features with traditional classification algorithms or neural network models. This method is time-consuming and laborious, so it is of great significance to explore an automatic computer-aided method for depression diagnosis.

To sum up, AGA patients have different degrees of depressive disorder. The meta heuristic algorithm has marked advantages in image recognition classification, but its classification accuracy needs to be further improved. In this study, a new depressive disorder recognition algorithm based on the meta heuristic algorithm was established and applied to AGA patients with depression, thereby evaluating the rehabilitation value of comprehensive psychological intervention for AGA patients, which can provide a reference for the diagnosis and treatment of AGA patients.

## Materials and Methods

### Research Objects and Grouping

54 patients with AGA who were treated in the Medical Cosmetic Center of Tongji Hospital from January 2020 to October 2020 were selected as the research objects, and all agreed to receive treatment in this hospital for a long time. Among them, there were 31 males and 23 females. Besides, they were 18–60 years old, and the average age was 39.15 ± 4.07 years. The criteria for inclusion were defined to include patients who were older than or equaled to 18 years old, and conformed to AGA diagnostic criteria. The criteria for exclusion were defined to include patients who suffered from hair loss caused by resting period, physiological and postpartum hair loss, and other cause, had neuropsychiatric diseases, and were accompanied with other serious systemic diseases. In addition, they were grouped into the control group (*n* = 27) and the intervention group (*n* = 27) based on the different ways of psychological intervention. The process was approved by the ethics committee of Tongji Hospital, and all the research objects included in this study signed the informed consent forms.

### Feature Selection Method Based on Meta Heuristic Algorithm

The optimization mathematical model of meta heuristic algorithm can be expressed as follows.

$$\begin{array}{l} \min f\left(x\right),\\ s.t.g_{i}\left(x\right)=0,\\ i=1,2,L,m;\\ h_{j}\left(x\right)\geq 0,\\ j=1,2,L,n. \end{array}$$ (1)

In the Eq. 1, x stands for the decision variable, representing the p-dimensional vector, and its calculation method is $x={\left\{x_{1},x_{2},\cdots ,x_{p}\right\}}^{T}\in {\mathbb{R}}^{p}$ . Besides, f(x) means the objective function, $g_{i}\left(x\right)$ indicates the equality constraint function, $h_{j}\left(x\right)$ represents the inequality constraint function, and s.t. expresses the abbreviation of “subject to,” which means “restricted to.”

The Filter algorithm in feature selection has a fast calculation speed, and the Wrapper algorithm has a higher calculation accuracy (Padfield et al., 2019). In this study, the Filter and Wrapper algorithms were combined to form a new feature selection algorithm, which was named FAW-FS. The two algorithms of analysis of variance (ANOVA) and mutual information were adopted to calculate the data to filter out the feature subset, thereby obtaining the union as the new feature space.

ANOVA (Peng et al., 2020) is a common special statistical hypothesis testing model in data analysis. The total variance (TV), total variance between groups (BGV), and variance within groups (WGV) of ANOVA are expressed as the following equations.

$$\mathrm{TV}={\sum_{\text{i}}\sum_{j}\left(Y_{ij}-\overset{-}{Y_{i}}\right)}^{2}$$ (2)

$$\mathrm{BGV}=\sum_{i}n_{i}{\left(\overset{-}{Y_{i}}-\overset{-}{Y_{t}}\right)}^{2}$$ (3)

$$\mathrm{WGV}={\sum_{i}\sum_{j}\left(Y_{ij}-\overset{-}{Y_{i}}\right)}^{2}$$ (4)

In the Eqs 2–4, *i* represents the group, and i=1,2,⋯,a ; $Y_{ij}$ means the *j*-th eigenvalue in the *i*-th dimension feature; *j* stands for the subscript of the observation value; $\overset{-}{Y_{t}}$ expresses the mean of all eigenvalues; $n_{i}$ represents the total number of the *i*-th dimensional eigenvalues; $\overset{-}{Y_{i}}$ indicates the mean of the *i*-th dimensional eigenvalues.

The mean square between groups (MSG) and mean square within groups (MSW) of ANOVA can be calculated as follows.

$$\mathrm{MSG}=\frac{\mathrm{BGV}}{k-1}=\frac{\sum_{i}n_{i}{\left(\overset{-}{Y_{i}}-\overset{-}{Y_{t}}\right)}^{2}}{k-1}$$ (5)

$$\mathrm{MSW}=\frac{\mathrm{WGV}}{N-k}=\frac{{\sum_{\text{i}}\sum_{j}\left(Y_{ij}-\overset{-}{Y_{i}}\right)}^{2}}{N-k}$$ (6)

In the Eq. 5 and Eq. 6, k and N stand for the dimension of the feature and the total number of eigenvalues, respectively.

Mutual information (MI) is mainly used to evaluate the joint probability distribution and marginal probability distribution between two variables (Wen et al., 2020). For discrete random variables, MI is defined as the following.

$$I\left(X;Y\right)=\sum_{y\in Y}\sum_{x\in X}p\left(x,y\right)\log\left(\frac{p\left(x,y\right)}{p\left(x\right)p\left(y\right)}\right)$$ (7)

In the Eq. 7, p(x,y) represents the joint probability distribution function between the two variables X and Y, p(x) means the marginal probability distribution of *X*, and p(y) shows the marginal probability distribution of *Y*.

For continuous random variables, MI can be defined as the following.

$$I\left(X;Y\right)=\int_{Y}\int_{X}p\left(x,y\right)\log\left(\frac{p\left(x,y\right)}{p\left(x\right)p\left(y\right)}\right)dxdy$$ (8)

In the Eq. 8, p(x,y) is the joint probability density function between the two variables *X* and *Y*, p(x) is the marginal probability density function of *X*, and p(y) is the marginal probability density function of *Y*.

Search strategy is the core of Wrapper’s selection method. In this study, the simulated annealing algorithm was introduced in the optimization process to improve the convergence of the Wrapper method and form a new genetic algorithm (GA). The simulated annealing algorithm can be expressed as follows.

$$p=\{\begin{array}{cc} 1, & ife_{1}<e_{2}\\ e^{-\frac{E\left(e_{1}-e_{2}\right)}{T}}, & ife_{1}>e_{2} \end{array}$$ (9)

In the Eq. 9, *T*, *p*, *E*, $e_{1}$ , and $e_{2}$ represent the temperature, the substitution probability, the internal energy, the objective function, and the objective function of the substitute object in turn.

Fitness is an important index to evaluate individual survivability in GA (Hasserjian, 2019). For the evaluation function f(x) , the fitness (*Fit*) function is F(x) , so the *Fit* of the individual *x* can be expressed as Fit=F[f(x)] . When the largest problem is solved, the *Fit* can be expressed as shown in the Eq. 10. What’s more, D means the minimum estimate of f(x) .

$$Fit=F\left[f\left(x\right)\right]=\{\begin{array}{cc} f\left(x\right)-D, & f\left(x\right)>D\\ 0, & f\left(x\right)\leq D \end{array}$$ (10)

**FIGURE 1:**
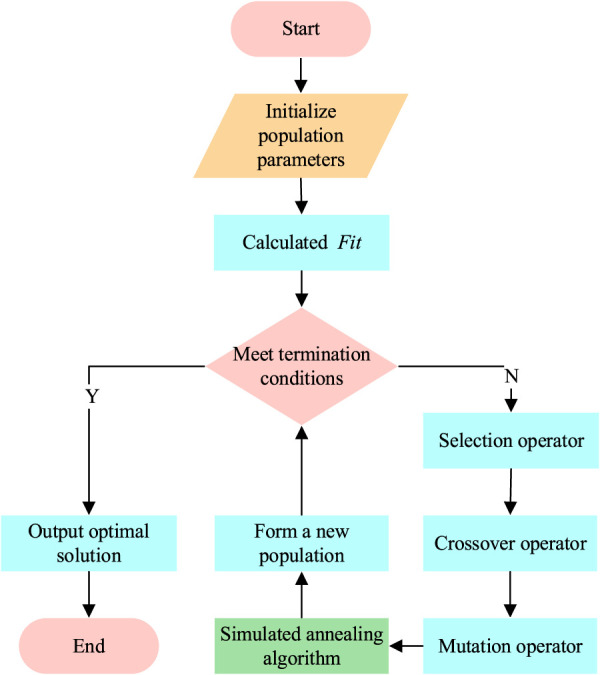
Flow chart of optimized GA.

After introducing the simulated annealing algorithm, GA is improved and optimized. For the optimized GA, the parameters should be set, including the number of iterations of the population, the number of local search iterations, the initial size, the crossover probability, the probability of mutation, and the temperature. Multiple suitable individuals are used as the initial population, and the fitness of individuals in the population is calculated. If the termination condition is satisfied and the output optimal solution satisfies the termination condition, the algorithm ends. For the individuals that do not meet the termination conditions, crossover operation is carried out for each pair of matching individuals in the population according to the specified selection operator, and new populations are generated according to the local search strategy. Then, it is further verified whether the individuals meet the termination conditions and enter the next cycle. The optimized GA flow chart is shown in Figure 1.

### Establishment of Depression Recognition Method Based on Meta Heuristic Algorithm

During the EEG acquisition process, different types of noise will have a certain impact on EEG. In this study, a band-pass filter is used to filter the data, and the EEG data are removed by the combination of Kalman filter and wavelet transform (Cabrera et al., 2018). The power spectrum entropy in nonlinear features is mainly applied to evaluate the strength of brain activity (Mendez-Balbuena et al., 2018). For the signal X(w) , the kilometer density is obtained after processing by the FAW-FS algorithm, and its power spectrum entropy can be expressed in the Eq. 11.

$$H_{w}=-\sum_{i=o}^{n}p_{x}\left(w_{i}\right)\log_{2}\left[p_{x}\left(w_{i}\right)\right]$$ (11)

Shannon entropy is employed to quantify EEG, and its calculation method is presented in the Eq. 12.

$$H_{x}=-x\log_{2}\left(x\right)-\left(1-x\right)\log_{2}\left(1-x\right)$$ (12)

The correlation dimension is mainly applied to describe the irregularity of EEG, and its calculation method is shown in the Eq. 13. In addition, ln⁡C(r) stands for the correlation function.

$$C=\underset{r\to 0}{\lim}\frac{\ln C\left(\text{r}\right)}{\ln r}$$ (13)

Kolmogorov entropy describes the dynamic characteristics and signal complexity of the signal. The larger the Kolmogorov entropy, the more chaotic the dynamic characteristics, and the more complex the signal (Mutanen et al., 2018). The Kolmogorov calculation can be expressed in the Eq. 14.

$$K=\underset{T\to 0}{\lim}\underset{\varepsilon \to 0}{\lim}\underset{N\to \infty }{\lim}\frac{1}{NT}\sum_{n=0}^{N-1}\left(K_{n+1}-K_{\text{n}}\right)$$ (14)

*C0* complexity is adopted to evaluate the degree of randomness of EEG, which can be calculated in the Eq. 15. The greater the *C0* complexity value, the stronger the randomness of the EEG sequence.

$$C0=\frac{\sum_{n=1}^{N}{\left|X\left(\text{n}\right)-Y\left(\text{n}\right)\right|}^{2}}{\sum_{n=1}^{N}{\left|X\left(\text{n}\right)\right|}^{2}}$$ (15)

In the above equation, X(n) represents the original EEG sequence, and Y(n) means the EEG sequence after Fourier transform.

**FIGURE 2:**
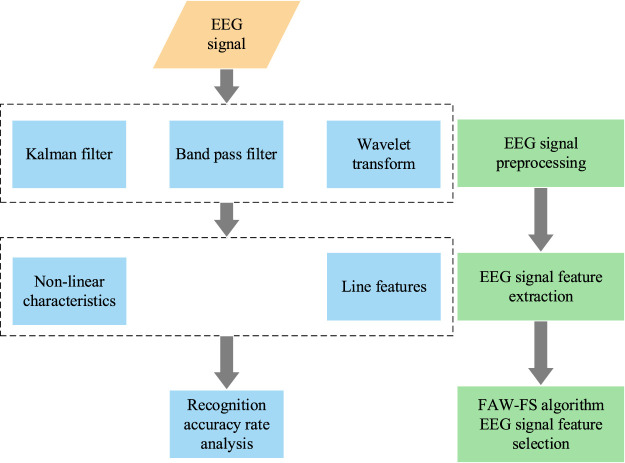
Flow chart of depression recognition based on meta heuristic algorithm.

The collected EEG data are used for filtering and electro-oculogram operation through the band-pass filter, Kalman filter, and wavelet transform. Then, the current and nonlinear characteristic EEG data are extracted, and finally, the FAW-FS algorithm is employed to select the EEG features. The flow chart of depression recognition based on meta heuristic algorithm is shown in Figure 2 below.

For each feature vector set output from the feature vector input module, it was first divided into a training set and a test set. Samples of the training set were derived from the public data set, with a sample size of 128. Each training set was divided into 1–5 of the 5 training subsets. Four training subsets out of the five training subsets were used for training in the deep forest each time, and the remaining one training subset was used as the verification set to verify the sub-model of training. The above sub-training process was repeated until every training subset in the whole training process made a verification set. After each verification of the trained sub-model, a set of feature vectors with a size of 12 was eventually obtained.

### Assessment Method for Classification Accuracy of FAW-FS Algorithm

The data sets published in the public database (http://archive.ics.uci.edu/ml/index.php) were compared with the FAW-FS algorithm established in this study to verify the classification accuracy of the FAW-FS algorithm. The information of the 7 public data sets selected in this study was displayed in Table 1.

**TABLE 1:** Relevant information of the public data sets.

Data set	Abbreviation	Sample size	Feature dimension	Class
Breast Cancer Wisconsin Data Set	BCW	569	30	2
Ionosphere Dataset	ION	351	33	2
Cryotherapy	CRY	90	7	2
SPECT Heart Data Set	SPE	267	22	2
Glass Identification	GI	241	9	2
Parkinson Multiple Sound Recording	PMS	1040	26	2
Connectionist Bench Data Set	CBD	208	60	2

Accuracy was employed to evaluate the recognition results of depression EEG signals, and its specific calculation method was shown in the following equation.

$$\mathrm{Accuracy}=\frac{\left|X:X\in D_{\text{t}}\cap \overset{-}{Y}\left(X\right)=Y\left(X\right)\right|}{\left|X:X\in D_{\text{t}}\right|}$$ (16)

In the Eq. 16, $D_{\text{t}}$ stood for the test data, X was the test sample, Y(X) represented the real classification result of the test sample, and $\bar{Y}\left(X\right)$ indicated the classification result identified by the classification model.

### Psychological Intervention Methods for Depression in Androgenic Alopecia Patients

The patients from both groups were given with paroxetine tablets (20 mg/time.d) for 3 weeks of drug treatment. On the basis of drug treatment, the control group was treated with routine psychological interventions, including sports, interest development, music listening, and social activities. The intervention group underwent the comprehensive psychological nursing intervention on the basis of routine psychological intervention. The content of comprehensive psychological intervention mainly included the following. First, patients were guided to make psychological adjustments with psychological counseling, psychological care, and psychological support, so as to reduce their depression and build confidence in treatment. Second, the medical staff should explain the clinical manifestations, treatment, and prognosis of AGA to patients, thereby establishing a proactive cognitive model. Third, the medical staff needed to help patients establish support from family members and friends.

### Psychological Intervention Observation Indexes for AGA Patients

The conditions of patients from the two groups were scored through the self-rating anxiety scale (SAS), self-rating depression scale (SDS), and Hamilton depression scale (HAMD) before and after treatment, respectively. SAS consists of 20 items in 1 dimension, which is scored from 1 to 4 levels; 50–59 points is considered as mild anxiety, 60–69 points as moderate anxiety, and 70 or above points as severe anxiety (Yue et al., 2020). There are also 20 items in 1 dimension of SDS, which are rated from 1 to 4 levels; 50–59 points is classified as mild depression, 60–69 points as moderate depression, and more than 70 points as severe depressions (Zou et al., 2016). The 1–4 levels were applied in the scoring of HAMD, with a total score of more than 35 points classified as severe depression; a score of 20–34 points indicates mild or moderate depression, and 8–20 points indicates mild depression (Zhao et al., 2019).

The differences of SAS, SDS, and HAMD scores before and after treatment were compared between the two groups. Besides, the changes in the depressive symptoms, treatment compliance, and quality of life of patients from the two groups were observed before and after treatment. The efficacy of depressive symptoms was evaluated by Jang et al. (2019). After treatment, the patient’s HAMD score reduction rate was greater than 75%, which means that the patient was cured; 50% < HAMD score reduction rate ≤75% indicated that the efficacy was markedly effective; 25% < HAMD score reduction rate ≤50% showed effectiveness; HAMD score reduction rate was less than 25%, meaning that the efficacy was ineffective. In addition, the total effect included clinical recovery, marked effect, and effectiveness.

The method of Kraepelien et al. (2019) was referred to assess the compliance to the treatment of depression. Those who strictly followed the doctor’s advice during treatment were complete compliance; those who basically followed the doctor’s advice were basic compliance; those who often did not follow the doctor’s advice or interrupt the treatment were regarded as non-compliance. Total compliance contained complete compliance and basic compliance.

Referring to the method of Teles et al. (2018), the quality of life of patients was evaluated before and after intervention for depression, and GQOLI-74 was adopted to analyze the 4 sub-items of the patient’s body, psychology, society, and substance.

### Statistical Methods

The experimental data were processed by SPSS19.0 statistical software, and the measurement data were expressed as mean ± standard deviation ( $\bar{x}\pm s$ ). The count data were represented by percentage (%), and the χ^2^ test was used. In addition, *p* < 0.05 indicated that the difference was statistically substantial.

## Results

### Analysis of Classification Accuracy Based on Meta Heuristic Logistic Regression Algorithm

**FIGURE 3:**
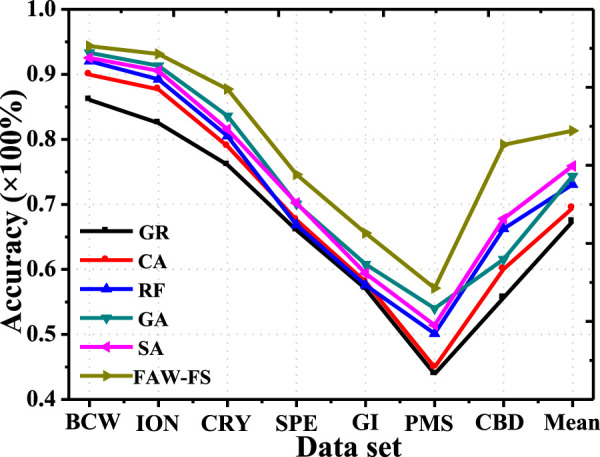
Comparison on the classification accuracy of different algorithms under the logistic regression feature selection method.

The classification accuracy of FAW-FS algorithm established in this study was compared with Correlation Attribute Eval (CA), Gain Ratio Attribute Eval (GR), Relief FAttribute Eval (RF), simulated annealing (SA) algorithm, and GA in the feature selection of logistic regression (Figure 3). In different public data sets, the classification accuracy of different algorithms changed in the same trend, while the classification accuracy of the same algorithm in different data sets varied greatly. In 7 different data sets, the classification accuracy of the FAW-FS algorithm was higher substantially than the accuracy of other algorithms, and its classification accuracy was 54.72–98.45%, with the mean classification accuracy of 80.87%.

**FIGURE 4:**
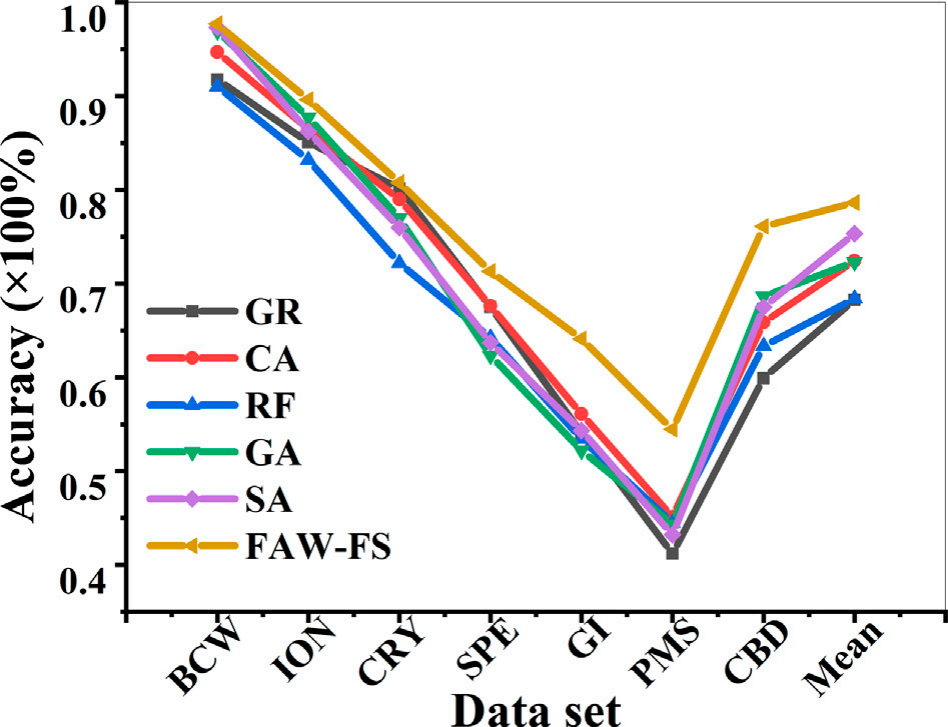
Comparison on the classification accuracy of different algorithms under the classification features of DT.

There was a comparison on the classification accuracy of the 6 algorithms under the classification features of DT (Figure 4). Among the 7 different data sets, all algorithms had the lowest classification accuracy in the PMS data set. The classification accuracy of FAW-FS algorithm rose obviously compared with other algorithms. Moreover, its classification accuracy was in the range of 43.28–98.81%, and the mean classification accuracy was 79.24%.

**FIGURE 5:**
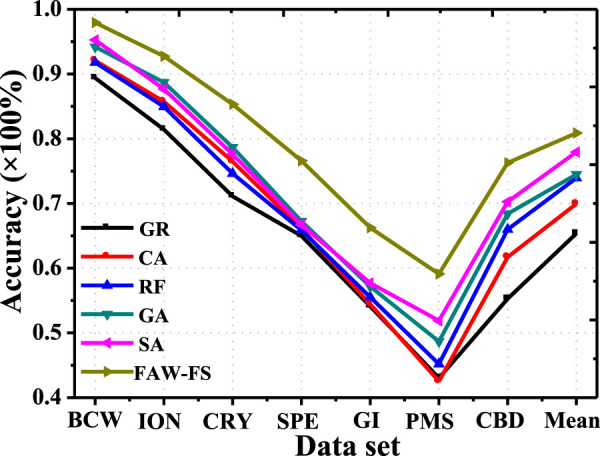
Comparison on the classification accuracy of different algorithms under the features of K-nearest neighbor algorithm.

The classification accuracy of the 6 algorithms was compared under the K-nearest neighbor algorithm (Figure 5). In the 7 different data sets, all algorithms had the highest classification accuracy in the BCW data set. The classification accuracy of FAW-FS algorithm elevated obviously in contrast to the accuracy of other algorithms. Its classification accuracy was distributed in the range of 42.94–99.12%, and the mean classification accuracy was 80.42%.

**FIGURE 6:**
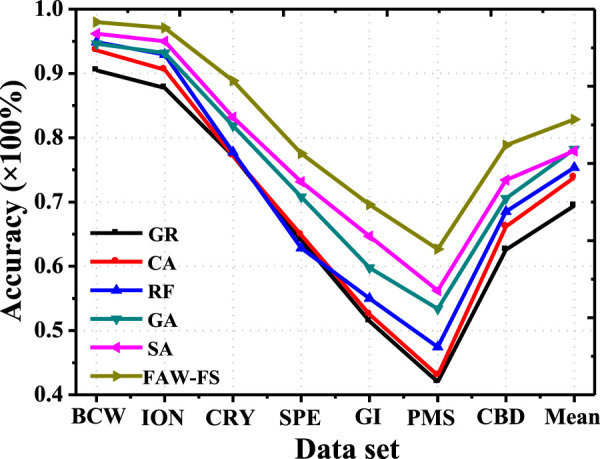
Comparison on the classification accuracy of different algorithms under the feature of SVM.

Figure 6 indicated that the classification accuracy of the 6 algorithms was compared under the features of SVM. In the 7 different data sets, the classification accuracy of the FAW-FS algorithm was higher hugely than that of other algorithms, and its classification accuracy was within 62.33–99.07%, with the mean classification accuracy of 83.07%.

**FIGURE 7:**
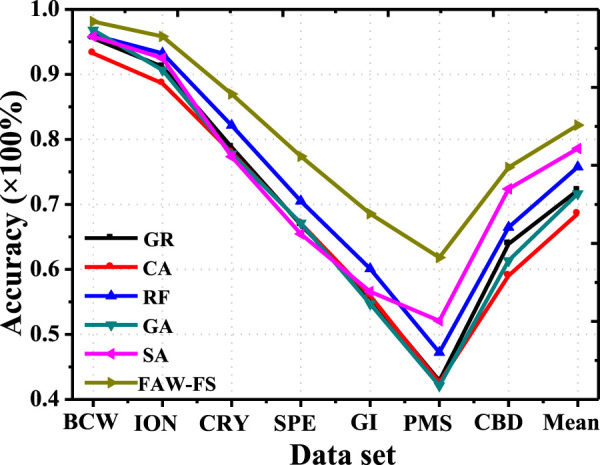
Analysis on accuracy based on meta heuristic algorithm under the feature of RF.

The classification accuracy of the six algorithms was compared under the characteristics of RF, and the results were presented in Figure 7. In the seven different data sets, the classification accuracy of the FAW-FS algorithm was higher greatly than the accuracy of other algorithms, and its classification accuracy was 61.93–99.26%, with the mean classification accuracy of 81.45%.

### Analysis of Electroencephalogram de-noising Results Based on Meta Heuristic Algorithm

**FIGURE 8:**
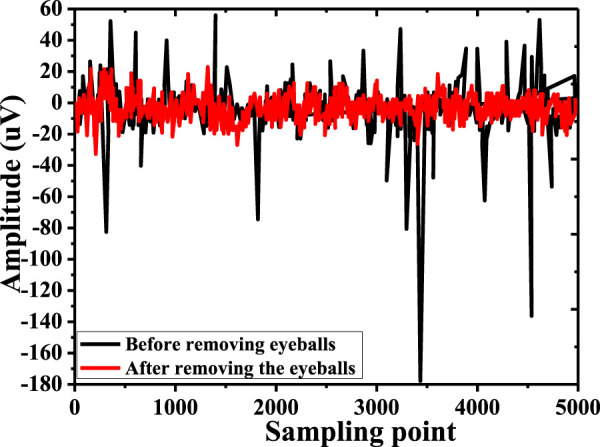
EEG before and after removing electro-oculogram noise.

In this study, a combination of Kalman filter and wavelet transform was used to preprocess EEG to remove electro-oculogram noise before the FAW-FS algorithm was adopted to extract and select EEG features, and the results were shown in Figure 8. Before electro-oculogram noise was removed, EEG had more electro-oculogram artifacts. After removing electro-oculogram noise, a pure EEG was obtained.

**FIGURE 9:**
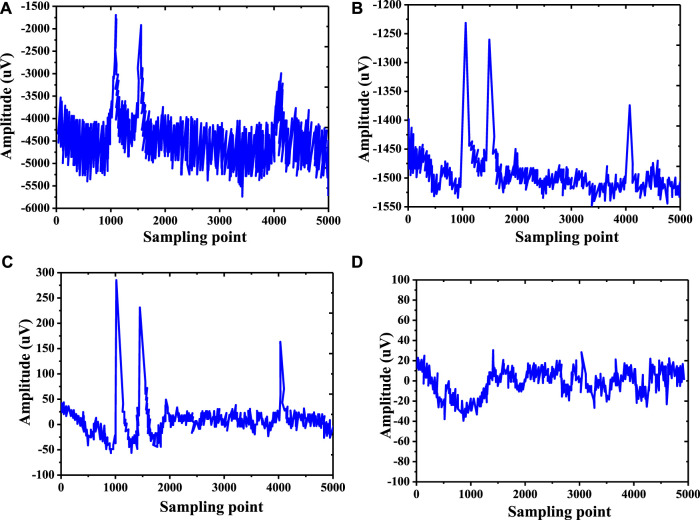
FAW-FS algorithm EEG de-noising results. [Note: **(A)**: Original EEG image; **(B)**: EEG image after down-sampling processing; **(C)**: EEG image after band-pass filter; **(D)**: EEG image of FAW-FS algorithm feature extraction].

During the processing of the EEG raw data (Figure 9A), the EEG data were initially processed with a down-sampling method of 1,000–250 Hz, so that the original signal was separated from the noise and the original data was enhanced (Figure 9B). A band-pass filter was applied to filter the data to remove the EEG artifacts in the EEG data (Figure 9C). Finally, the Kalman filter and wavelet transform were combined to remove the electro-oculogram artifacts in the EEG data, and the pure EEG data were obtained after extraction by the FAW-FS algorithm established in this study (Figure 9D).

### Analysis on the Accuracy of Electroencephalogram Feature Selection Based on Meta Heuristic Algorithm

**FIGURE 10:**
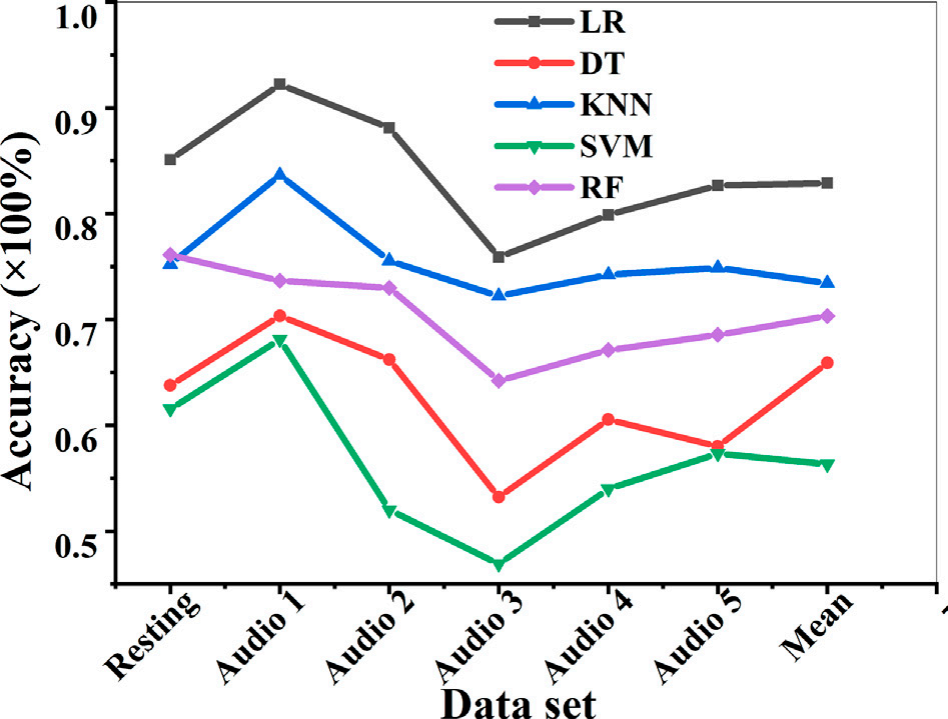
Analysis on the accuracy of EEG feature selection under different conditions.

The accuracy of EEG feature selection of the FAW-FS algorithm under different data sets was analyzed under the resting state and five audio stimuli, as shown in Figure 10. It was found that among the five classification algorithms, the LR algorithm had the highest feature selection accuracy under the five audio types, and its mean feature selection accuracy was 82.94%, followed by KNN (73.72%) and RF (70.09%). The mean accuracy of feature selection for DT and SVM was 65.77 and 55.49%, respectively. The mean accuracy of feature selection of SVM was the lowest among the 5 algorithms. What’s more, 5 different algorithms all had the highest mean accuracy of feature selection on audio stimulus 1 in the 6 data sets of resting state EEG and audio stimulation EEG, and the lowest accuracy was on audio stimulus 3.

### Analysis on the Accuracy of the Classification Algorithm on the Test Set

**FIGURE 11:**
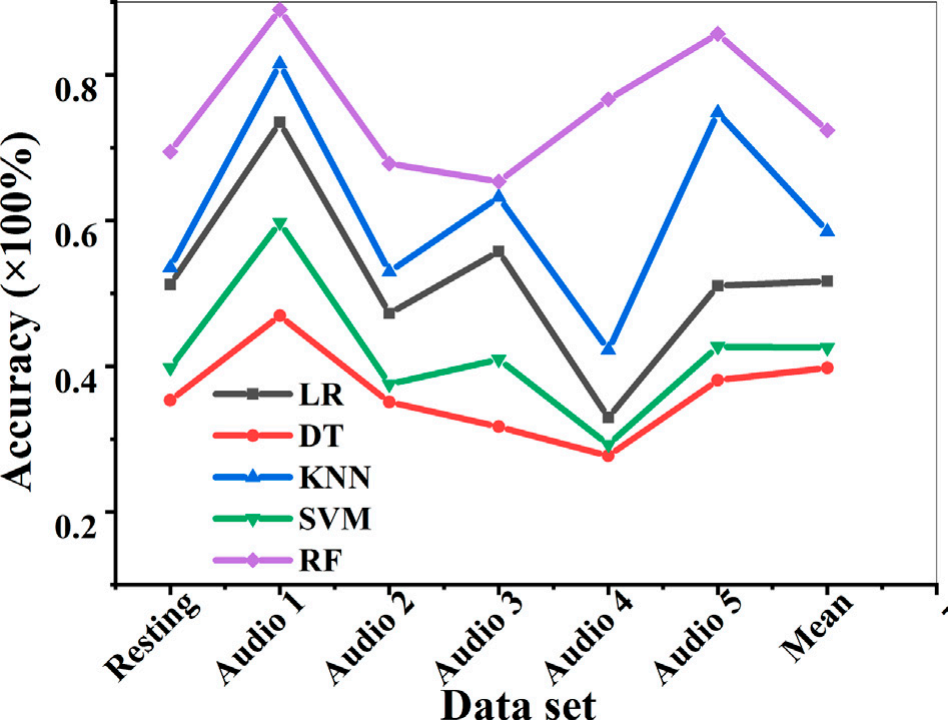
The accuracy analysis of the classification algorithm on the test set.

There was an analysis on the EEG accuracy of the FAW-FS algorithm under different data sets under the resting state and the five audio stimuli (Figure 11). It revealed that among the five classification algorithms, the RF algorithm had the highest classification accuracy under the five audio types, with a mean accuracy of 73.01%, followed by KNN (58.94%) and LR (52.76%). In addition, the mean accuracy of DT and SVM were 40.18 and 42.55% in turn. The mean accuracy of feature selection of DT was the lowest among the five algorithms. In the 6 data sets of resting state EEG and audio stimulation EEG, 5 different algorithms had the highest average accuracy on audio stimulation 1, and there was the lowest mean accuracy on audio stimulation 4.

### Comparison on Basic Data of Patients From the Two Groups

The basic data of patients from the two groups were compared and analyzed, and the results were displayed in Table 2. There was no statistical difference in age, gender ratio, body mass index (BMI), weight, height, and course of disease between the two groups (*p* > 0.05).

**TABLE 2:** Comparison on basic data of patients from the two groups.

Group	Control group (*n* = 27)	Intervention group (*n* = 27)	*t* value or χ^2^ value	*p* Value
Age (years old)	40.12 ± 4.07	38.85 ± 5.03	0.554	0.121
Male [cases, (%)]	15 (55.56)	16 (59.26)	0.083	0.224
Female [case, (%)]	12 (44.44)	11 (40.74)	0.063	0.257
BMI (kg/m^2^)	24.92 ± 2.07	26.03 ± 2.59	0.134	0.717
Weight (kg)	59.09 ± 11.98	62.07 ± 9.98	0.094	0.392
Height (cm)	162.95 ± 5.43	163.77 ± 5.19	0.141	0.762
Course of disease (years)	2.09 ± 1.28	2.26 ± 1.33	0.196	0.824

### Electroencephalogram Changes in Androgenic Alopecia Patients With Depression Before and After Psychological Intervention

**FIGURE 12:**
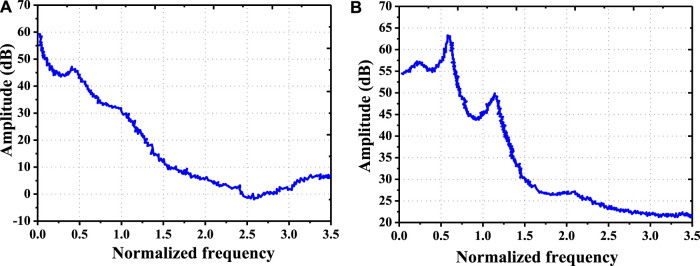
EEG changes in AGA patients with depression before and after psychological intervention. [Note: **(A)**: EEG of patients with AGA before psychological intervention; **(B)**: EEG of patients with AGA after psychological intervention].

The changes of EEG before and after psychological intervention in AGA patients with depression were analyzed (Figure 12). Before the intervention, the EEG power spectrum amplitude of AGA patients showed a smoothly downward trend with the continuous growth of the normalized frequency. The EEG power spectrum amplitude was distributed in the range of −0.5026–59.8248 dB, and the EEG mean power spectrum amplitude was 17.883 ± 8.190 dB. After the intervention, the EEG power spectrum amplitude of AGA patients rose first and then decreased with the continuous increase of the normalized frequency. The EEG power spectrum amplitude was distributed in the range of 21.0315–63.9881 dB, and the EEG mean power spectrum amplitude was 34.854 ± 3.465 dB.

### Comparison on Depression Improvement Between the Two Groups of Patients Before and After Psychological Intervention

**FIGURE 13:**
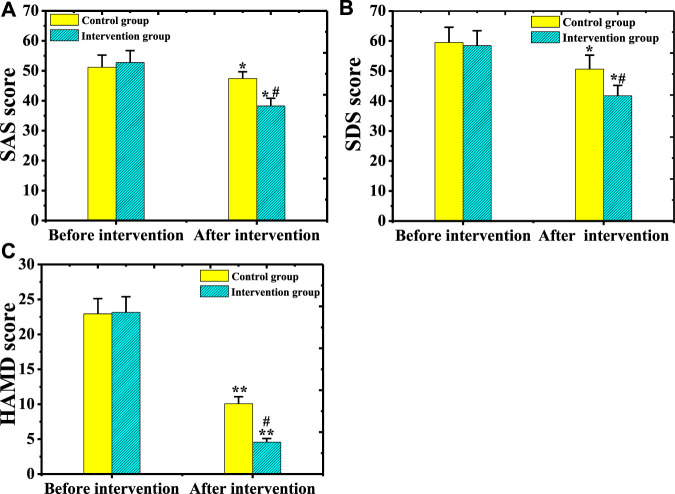
Comparison on SDS, SAS, and HAMD scale scores between the two groups of patients before and after psychological intervention.[Note: **(A)**: Comparison of SAS scale scores before and after psychological intervention between the two groups of patients; **(B)**: Comparison of SDS scale scores before and after psychological intervention between the two groups of patients; **(C)**: Comparison of HAMD scale scores before and after psychological intervention between the two groups of patients; * indicated *p* < 0.05 compared with before the intervention; ** meant *p* < 0.01 compared with before the intervention; # showed *p* < 0.05 compared with the control group].

The scores of SDS, SAS, and HAMD scales before and after psychological intervention between the two groups were compared (Figure 13). There was no marked difference in SDS, SAS, and HAMD scale scores between the two groups of patients before the intervention (*p* > 0.05). After the intervention, the SDS and SAS scores of patients from the two groups were lower steeply than those before the treatment, and the difference was statistically obvious (*p* < 0.05). After the intervention, the HAMD scale scores of patients from the two groups were dramatically different from those before the treatment (*p* < 0.01). The SDS, SAS, and HAMD scores of the intervention group reduced sharply in contrast to the scores of the control group (*p* < 0.05).

**FIGURE 14:**
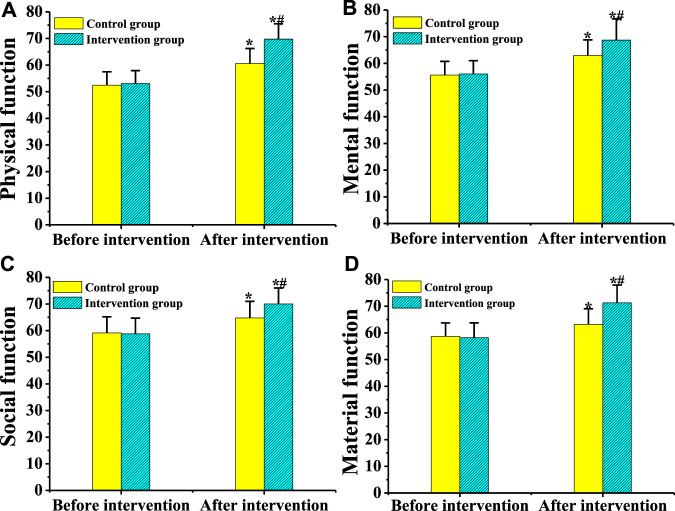
Comparison on the quality of life of patients from the two groups before and after psychological intervention.[Note: **(A)**: Comparison of the physical function of the two groups of patients before and after psychological intervention; **(B)**: Comparison of the psychological function of the two groups of patients before and after psychological intervention; **(C)**: Comparison of the social function of the two groups of patients before and after psychological intervention; **(D)**: Comparison of the substance function of the two groups of patients before and after psychological intervention; * indicated *p* < 0.05 compared with before the intervention; # showed that *p* < 0.05 in contrast to the control group].

There was a comparison on the quality of life scores of patients from the control group and the intervention group before and after psychological intervention (Figure 14). Before the intervention, there was no significant difference in the physical function, psychological function, social function, and substance function between the two groups of patients (*p* > 0.05). After the intervention, the physical function, mental function, social function, and substance function of patients from the two groups increased hugely compared with before the intervention, with a statistically huge difference (*p* < 0.05). The scores of physical function, mental function, social function, and substance function of the intervention group were higher markedly than the scores of the control group (*p* < 0.05).

### Comparison on the Depression Efficacy and Compliance of Patients From the Two Groups After Treatment

**FIGURE 15:**
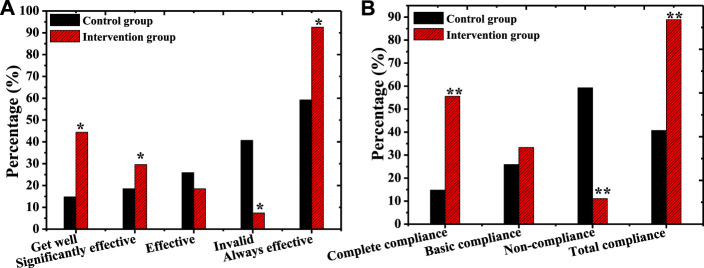
Comparison on the efficacy and compliance of depression after psychological intervention between the two groups.[Note: **(A)**: Comparison on the efficacy of depression after intervention between the two groups of patients; **(B)**: Comparison of intervention compliance between the two groups of patients; * indicated *p* < 0.05 compared with the control group; ** meant *p* < 0.01 compared with the control group].

Figure 15 showed the statistical analysis on the improvement of the efficacy of depression after treatment in patients from the two groups. In the control group, there were 4 cured cases (14.81%), 5 cases (18.52%) with marked effect, 7 effective cases (25.93%), and 11 cases (40.74%) with no effect after the intervention, and the total number of effective cases was 16 (59.26%). In the intervention group, 12 cases (44.44%) were cured, 8 cases (29.63%) were markedly effective, 5 cases (18.52%) were effective, and 2 cases (7.41%) were ineffective, so the total number of effective cases was 25 (92.59%). The proportion of cured, markedly effective, and total effective patients in the intervention group was higher greatly than the proportion of the control group (*p* < 0.05). After the intervention, there were 4 cases (14.81%) with complete compliance, 7 cases (25.93%) with basic compliance, and 16 cases (59.26%) with no compliance in the control group, and the total number of cases with compliance was 11 (40.74%). In the intervention group, 15 patients (55.56%), 9 patients (33.33%), and 3 patients (11.112%) were completely compliant, basically compliant, and non-compliant, so there were 24 cases with compliance (88.89%). The proportion of patients with complete compliance and total compliance in the intervention group elevated substantially compared with the control group, and there was a significant difference between the two (*p* < 0.01).

## Discussion

In this study, FAW-FS algorithm was established based on meta-heuristic algorithm and applied to depression disorder recognition. The classification accuracy of FAW-FS algorithm was compared with CA, GR, RF, SA, and GA algorithms in deep learning under different feature selection methods. CA, GR and RF algorithms are all classic Filter feature selection methods, and SA and GA algorithms are commonly used methods in Wrapper feature selection methods (Becerra-Sánchez et al., 2020). The results of this study showed that FAW-FS algorithm had the highest classification accuracy in LR, DT, KNN, SVM, and RF, which were 80.87, 79.24, 80.42, 83.07, and 81.45% respectively. These results indicated that the classification accuracy of FAW-FS established in this study was higher than that of the Filter feature selection method and Wrapper feature selection method. The reason was that the FAW-FS algorithm had the high accuracy of Wrapper and simplicity of Filter calculation (Albasri et al., 2019), and the two Filter feature selection algorithms through ANOVA and mutual information were used to initially screen the original data, forming a new feature space (Varsehi and Firoozabadi, 2021). In the calculation process, the local optimization method of individuals in the population and the simulated annealing strategy were adopted to improve the premature convergence of GA, and finally, the classification accuracy of the FAW-FS algorithm was promoted. The results of this study suggested that the LR algorithm had the highest feature selection accuracy of 82.94% under five audio types. The five different algorithms all had the highest average feature selection accuracy on audio stimulus 1, and audio stimulus 3 had the lowest. In this study, the EEG data on the three electrode positions Fp1, Fp2, and Fpz were used to identify depression, and the highest recognition accuracy was 73.01%. Mohammadi et al. (2020) (Mohammadi and Moradi, 2021) used meta-heuristic algorithm to classify FP1, FP2, and FPZ EEG data of patients with depression, and the highest accuracy was 70.24%. Bachmann et al. (2018) also applied Fp1, Fp2, and Fpz to classify depression, and the highest accuracy was 71.29%. The classification accuracy of the FAW-FS algorithm in this study was higher substantially than these studies, indicating that the fusion feature selection algorithm FAW-FS had a certain practicability and generalization, which could improve the accuracy of depression recognition to a certain extent.

At present, a large number of research results disclose that necessary psychological intervention for patients with AGA is of great significance for disease control and treatment (Wang et al., 2018). In this study, patients from the two groups were treated with different psychological intervention methods to explore the influence of comprehensive psychological intervention methods on patients. The results showed that the scores of SDS, SAS, and HAMD of patients from the two groups dropped hugely after intervention compared with before treatment (*p* < 0.05). The scores of SDS, SAS, and HAMD in the intervention group were lower dramatically than the scores of the control group (*p* < 0.05). The scores of physical function, psychological function, social function, and substance function in patients from the intervention group were higher remarkably than those of the control group (*p* < 0.05). The proportion of cured, effective, and total effective patients from the intervention group elevated obviously compared with the control group (*p* < 0.05). The proportion of patients with complete compliance and total compliance in the intervention group was bigger substantially than the proportion of the control group (*p* < 0.01). Therefore, these results showed that comprehensive psychological intervention was more helpful to the recovery of patients with AGA, which was similar to the research findings of Gonzalez et al. (2010).

## Conclusion

In this study, a depression EEG signal recognition model FAW-FS was established based on deep learning meta-heuristic algorithm, which was applied to the recognition of depression EEG signals in patients with AGA. A comprehensive psychological intervention method was adopted to intervene in patients with AGA. The results showed that the FAW-FS algorithm based on deep learning meta-heuristic algorithm could significantly improve the accuracy of depression disorder recognition, and comprehensive psychological intervention played a positive role in the rehabilitation of depression disorder in patients with AGA. However, there are still some shortcomings in this study. In this study, the EEG data on FP1, FP2, and FPZ are only classified, and the EEG characteristic data of Beta bands related to depression recognition are not analyzed. The value of FAW-FS algorithm in the classification of EEG characteristic data in the sub-bands will be further explored in the future work. In conclusion, the FAW-FS algorithm established based on meta-heuristic algorithm in this study can improve the accuracy of EEG signal recognition for depression disorders, and comprehensive psychological intervention plays a positive role in the rehabilitation of depression disorders in patients with AGA, thereby providing a reference basis for the diagnosis and treatment of AGA patients.

## Data Availability

The original contributions presented in the study are included in the article/Supplementary Material, further inquiries can be directed to the corresponding author.

## References

[B1] Albasri A. Abdali-Mohammadi F. Fathi A. (2019). EEG Electrode Selection for Person Identification Thru a Genetic-Algorithm Method. J. Med. Syst. 43 (9), 297. 10.1007/s10916-019-1364-8 31350595

[B2] Bachmann M. Päeske L. Kalev K. Aarma K. Lehtmets A. Ööpik P. (2018). Methods for Classifying Depression in Single Channel EEG Using Linear and Nonlinear Signal Analysis. Computer Methods Programs Biomed. 155, 11–17. 10.1016/j.cmpb.2017.11.023 29512491

[B3] Becerra-Sánchez P. Reyes-Munoz A. Guerrero-Ibañez A. (2020). Feature Selection Model Based on EEG Signals for Assessing the Cognitive Workload in Drivers. Sensors 20 (20), 5881. 10.3390/s20205881 PMC7589097 33080866

[B4] Cabrera A. F. Petersen E. B. Graversen C. Sorensen A. T. Lunner T. Rank M. L. (2018). Individual Classification of Single Trial EEG Traces to Discriminate Brain Responses to Speech with Different Signal-To-Noise Ratios. Annu. Int. Conf. IEEE Eng. Med. Biol. Soc. 2018, 987–990. 10.1109/EMBC.2018.8512491 30440556

[B5] Craik A. He Y. Contreras-Vidal J. L. (2019). Deep Learning for Electroencephalogram (EEG) Classification Tasks: a Review. J. Neural Eng. 16 (3), 031001. 10.1088/1741-2552/ab0ab5 30808014

[B6] Eilbeigi E. Setarehdan S. K. (2018). Global Optimal Constrained ICA and its Application in Extraction of Movement Related Cortical Potentials from Single-Trial EEG Signals. Computer Methods Programs Biomed. 166, 155–169. 10.1016/j.cmpb.2018.07.013 30415714

[B7] Gonzalez M. E. Cantatore-Francis J. Orlow S. J. (2010). Androgenetic Alopecia in the Paediatric Population: a Retrospective Review of 57 Patients. Br. J. Dermatol. 163 (2), 378–385. 10.1111/j.1365-2133.2010.09777.x 20346026

[B8] Hasserjian R. P. (2019). Myelodysplastic Syndrome Updated. Pathobiology 86 (1), 7–13. 10.1159/000489702 30041243

[B9] Jang H.-M. Lee K.-E. Kim D.-H. (2019). The Preventive and Curative Effects of Lactobacillus Reuteri NK33 and Bifidobacterium Adolescentis NK98 on Immobilization Stress-Induced Anxiety/depression and Colitis in Mice. Nutrients 11 (4), 819. 10.3390/nu11040819 PMC6521032 30979031

[B10] Kraepelien M. Blom K. Lindefors N. Johansson R. Kaldo V. (2019). The Effects of Component-specific Treatment Compliance in Individually Tailored Internet-Based Treatment. Clin. Psychol. Psychother 26 (3), 298–308. 10.1002/cpp.2351 30650232 PMC6635903

[B11] Lake F. (2019). From Industry 4.0 to Lab 4.0. Biotechniques 66 (6), 247. 10.2144/btn-2019-0061 31134818

[B12] Lolli F. Pallotti F. Rossi A. Fortuna M. C. Caro G. Lenzi A. (2017). Androgenetic Alopecia: a Review. Endocrine 57 (1), 9–17. 10.1007/s12020-017-1280-y 28349362

[B13] Mendez-Balbuena I. Arrieta P. Huidobro N. Flores A. Lemuz-Lopez R. Trenado C. (2018). Augmenting EEG-Global-Coherence with Auditory and Visual Noise. Medicine (Baltimore) 97 (35), e12008. 10.1097/MD.0000000000012008 30170407 PMC6393074

[B14] Mohammadi Y. Moradi M. H. (2021). Prediction of Depression Severity Scores Based on Functional Connectivity and Complexity of the EEG Signal. Clin. EEG Neurosci. 52 (1), 52–60. 10.1177/1550059420965431 33040603

[B15] Munoz R. Olivares R. Taramasco C. Villarroel R. Soto R. Barcelos T. S. (2018). Using Black Hole Algorithm to Improve EEG-Based Emotion Recognition. Comput. Intell. Neurosci. 2018, 3050214. 10.1155/2018/3050214 29991942 PMC6016227

[B16] Mutanen T. P. Metsomaa J. Liljander S. Ilmoniemi R. J. (2018). Automatic and Robust Noise Suppression in EEG and MEG: The SOUND Algorithm. Neuroimage 166, 135–151. 10.1016/j.neuroimage.2017.10.021 29061529

[B17] Padfield N. Zabalza J. Zhao H. Masero V. Ren J. (2019). EEG-based Brain-Computer Interfaces Using Motor-Imagery: Techniques and Challenges. Sensors 19 (6), 1423. 10.3390/s19061423 PMC6471241 30909489

[B18] Peng G. Nourani M. Harvey J. Dave H. (2020). Feature Selection Using F-Statistic Values for EEG Signal Analysis. Annu. Int. Conf. IEEE Eng. Med. Biol. Soc. 2020, 5963–5966. 10.1109/EMBC44109.2020.9176434 33019330

[B19] Phadikar S. Sinha N. Ghosh R. (2021). Automatic Eyeblink Artifact Removal from EEG Signal Using Wavelet Transform with Heuristically Optimized Threshold. IEEE J. Biomed. Health Inform. 25 (2), 475–484. 10.1109/JBHI.2020.2995235 32750902

[B20] Rajabi F. Drake L. A. Senna M. M. Rezaei N. (2018). Alopecia Areata: a Review of Disease Pathogenesis. Br. J. Dermatol. 179 (5), 1033–1048. 10.1111/bjd.16808 29791718

[B21] Starace M. Orlando G. Alessandrini A. Piraccini B. M. (2020). Female Androgenetic Alopecia: an Update on Diagnosis and Management. Am. J. Clin. Dermatol. 21 (1), 69–84. 10.1007/s40257-019-00479-x 31677111

[B22] Tanaka Y. Aso T. Ono J. Hosoi R. Kaneko T. (2018). Androgenetic Alopecia Treatment in Asian Men. J. Clin. Aesthet. Dermatol. 11 (7), 32–35. PMC6057731 30057663

[B23] Teles F. Amorim de Albuquerque A. L. Freitas Guedes Lins I. K. Carvalho Medrado P. Falcão Pedrosa Costa A. (2018). Quality of Life and Depression in Haemodialysis Patients. Psychol. Health Med. 23 (9), 1069–1078. 10.1080/13548506.2018.1469779 29706105

[B24] Varsehi H. Firoozabadi S. M. P. (2021). An EEG Channel Selection Method for Motor Imagery Based Brain-Computer Interface and Neurofeedback Using Granger Causality. Neural Networks 133, 193–206. 10.1016/j.neunet.2020.11.002 33220643

[B25] Völker J. M. Koch N. Becker M. Klenk A. (2020). Caffeine and its Pharmacological Benefits in the Management of Androgenetic Alopecia: a Review. Skin Pharmacol. Physiol. 33 (3), 93–109. 10.1159/000508228 32599587

[B26] Wang X. Xiong C. Zhang L. Yang B. Wei R. Cui L. (2018). Psychological Assessment in 355 Chinese College Students with Androgenetic Alopecia. Medicine (Baltimore) 97 (31), e11315. 10.1097/MD.0000000000011315 30075498 PMC6081179

[B27] Wen D. Yuan J. Zhou Y. Xu J. Song H. Liu Y. (2020). The EEG Signal Analysis for Spatial Cognitive Ability Evaluation Based on Multivariate Permutation Conditional Mutual Information-Multi-Spectral Image. IEEE Trans. Neural Syst. Rehabil. Eng. 28 (10), 2113–2122. 10.1109/TNSRE.2020.3018959 32833638

[B28] Yue T. Li Q. Wang R. Liu Z. Guo M. Bai F. (2020). Comparison of Hospital Anxiety and Depression Scale (HADS) and Zung Self-Rating Anxiety/Depression Scale (SAS/SDS) in Evaluating Anxiety and Depression in Patients with Psoriatic Arthritis. Dermatology 236 (2), 170–178. 10.1159/000498848 31434087

[B29] Zhao B. Li Z. Wang Y. Ma X. Wang X. Wang X. (2019). Can Acupuncture Combined with SSRIs Improve Clinical Symptoms and Quality of Life in Patients with Depression? Secondary Outcomes of a Pragmatic Randomized Controlled Trial. Complement. Therapies Med. 45, 295–302. 10.1016/j.ctim.2019.03.015 31331577

[B30] Zou Q. Jiang Y. Mu F. Shi Y. Fang Y. (2016). Correlation of Axial Spondyloarthritis with Anxiety and Depression. Med. Sci. Monit. 22, 3202–3208. 10.12659/msm.897232 27611598 PMC5021017

